# Bridging the Gap between Cognitive Behaviour Therapy and Psychodynamic Therapy: The Added Value and Impact of Introducing Training in Cognitive Analytic Therapy

**DOI:** 10.1192/j.eurpsy.2023.805

**Published:** 2023-07-19

**Authors:** F. Cassar, D. Mamo

**Affiliations:** Psychiatry, Mental Health Services Malta, Attard, Malta

## Abstract

**Introduction:**

Cognitive Analytic therapy (CAT) is a brief focal therapy consisting of 16 - 24 sessions in the context of complex cases. It involves three stages: reformulation, recognition and revision (Taylor *et al*, 2017 Dec;90(4):511-529). CAT was shown to have an effect within a 24-session format and has been found to be particularly helpful by those who work with these ‘hard to help’ patients, including abuse survivors, the elderly and offenders.

**Objectives:**

To introduce CAT to Malta as a Creative Service Initiative with outcome measures in part-fulfillment of the Malta Postgraduate Training Programme in Psychiatry. To perform a qualitative study to see the attitudes of course participants towards other modalities of therapy besides Cognitive Behavioral Therapy and Psychodynamic Psychotherapy.

**Methods:**

A CAT Skills Training Course was organised for the first time in Malta in collaboration with Richmond Foundation, the Malta Association of Psychiatrists and the International Cognitive Analytic Therapy Association. The course was delivered over 6 days. This was divided into 35 hours of theory and 8 hours of skills based sharing and learning with a particular emphasis on contextual mapping including team dynamics, systemic and structural role positioning within health services and orgnaistaions.

A qualitative questionnaire was disseminated to participants at the end of the 6-month programme which included supervision via skype and completion of a reflective essay. A follow-up training course in 2022 was organised and the introduction of CAT Malta to psychiatric trainees planned for March 2023.

**Results:**

A total of 20 participants participated in the original CAT Training in March 2020 of which 1 was a psychiatrist, 2 were psychiatric trainees, 6 were social workers, 3 were counsellors and the remainder were psychologists. From this group of participants 4 members (1 psychiatrist, 1 psychiatric trainee, one psychologist and one social worker) continued to level 2 of training with the aim of continuing to CAT practitioner training. The remaining 16 participants dropped out in view of personal commitments and pressures presented during the COVID-19 pandemic. A new cohort of 14 participants were recruited in September 2022 of which all plan to continue to level 2 of training with the hopes of becoming CAT practitioners. A qualitative study into their reflective essays is being undertaken.

CAT Malta was established in 2022 with the 4 members who continued CAT training level 2. These members are in the process of becoming CAT practitioners and pioneering this new treatment into mental health services amongst the Maltese islands.

**Conclusions:**

In conclusion the above proves that the implementation of CAT Training is feasible and acceptable. It will be introduced to Maltese psychiatric trainees in March 2023.

**Disclosure of Interest:**

None Declared

